# Protective Effects of Benzoic Acid,* Bacillus *Coagulans, and Oregano Oil on Intestinal Injury Caused by Enterotoxigenic* Escherichia coli* in Weaned Piglets

**DOI:** 10.1155/2018/1829632

**Published:** 2018-08-27

**Authors:** Junning Pu, Daiwen Chen, Gang Tian, Jun He, Ping Zheng, Xiangbing Mao, Jie Yu, Zhiqing Huang, Ling Zhu, Junqiu Luo, Yuheng Luo, Bing Yu

**Affiliations:** ^1^Key Laboratory of Animal Disease-Resistance Nutrition, Animal Nutrition Institute, Sichuan Agricultural University, 46# Xinkang Road, Yucheng District, Yaan, Sichuan 625014, China; ^2^Key Laboratory of Animal Biotechnology Center of Sichuan Province, College of Veterinary Medicine, Sichuan Agricultural University, Chengdu, Sichuan, 611134, China

## Abstract

The use of antibiotics as growth promoters in feed has been fully or partially banned in several countries. The objective of this study was to investigate the effects of benzoic acid (A),* bacillus *coagulans (B) and oregano oil (O) combined supplementation on growth performance and intestinal barrier in piglets challenged with enterotoxigenic* Escherichia coli* (ETEC). Thirty piglets were randomly assigned to 6 treatments: (1) nonchallenged control (CON); (2) ETEC-challenged control (ETEC); (3) antibiotics + ETEC (AT); (4) A + B + ETEC (AB); (5) A + O + ETEC (AO); (6) A + B + O + ETEC (ABO). On day 22, piglets were orally challenged with ETEC or saline. The trial lasted 26 days. Dietary AO and ABO inhibited the reduction of growth performance and the elevation of diarrhoea incidence in piglets induced by ETEC (*P*<0.05). AB, AO, and ABO prevented the elevation of serum TNF-*α* and LPS concentrations in piglets induced by ETEC (*P*<0.05). ABO alleviated the elevation of TNF-*α* and IL-1*β* concentrations and the reduction of sIgA level in jejunal mucosa induced by ETEC (*P*<0.05). Furthermore, ABO upregulated mRNA expressions of Claudin-1 and Mucin2 (*P*<0.05), downregulated mRNA abundances of TLR4 and NOD2 signaling pathways related genes in jejunal mucosa (*P*<0.05), and improved the microbiota in jejunal and cecal digesta (*P*<0.05) compared with ETEC group. These results indicated that benzoic acid,* bacillus *coagulans, and oregano oil combined supplementation could improve growth performance and alleviate diarrhoea of piglets challenged with ETEC via improving intestinal mucosal barrier integrity, which was possibly associated with the improvement of intestinal microbiota and immune status. The combination of 3000 g/t benzoic acid + 400 g/t* bacillus* coagulans + 400 g/t oregano oil showed better effects than other treatments in improving growth performance and intestinal health of piglets, which could be used as a viable substitute for antibiotic.

## 1. Introduction

Enterotoxigenic* Escherichia coli *(ETEC) infection is a major pathogenic cause of gastroenteritis and diarrhoea in children and young animals [1, 2]. Recent studies have also shown that ETEC infection could impair intestine and induce the inflammatory response in children and weaned piglets [3, 4]. To control enteric infections, antibiotics have been widely used in animal production as growth promoters and therapeutic medicines [5]. However, the overuse of antibiotics has caused lots of problems, such as drug resistance, environmental pollution, antibiotic-residues in animal products, and inhibition of innate immune function [6, 7]. Thus, considering both the safety of the consumer and the profitability for the farmer, alternatives to antibiotics are desperately needed.

In recent years, natural alternatives for feed antibiotics with organic acids and their salts, essential oils, probiotics, prebiotics, and oligosaccharides have received much attention due to their antibacterial activities in the gastrointestinal tract of livestock and poultry [8, 9]. Some researchers have proposed that organic acid could improve growth performance and has antibacterial action primarily via decreasing pH values of the stomach and gut digesta, modulating microbial populations, improving nutrients digestion and other possible mechanisms [10, 11]. Benzoic acid, as a kind of organic acid, was authorized to be used in pigs at the dose of 0.5% to 1.0% by European Union in 2003. Previous studies indicated that benzoic acid could improve the growth performance and nutrient digestibility, inhibit the proliferation of pathogenic bacteria, and maintain intestinal microecological balance [12, 13].The term probiotics has been defined as live microbial cell preparations or microbial cell components feed supplements, which beneficially improve growth performance by maintaining intestinal microbial balance and stimulating immune response of animals [14, 15].* Bacillus* coagulans, as a kind of probiotic, not only has all the characteristics of lactic acid bacteria but also has strong resistance to acid, high temperature, high pressure, and easy storage properties. Some researchers have proposed that* bacillus* coagulans could improve the growth performance [16], maintain intestinal microecological balance [17], and improve immune response of animals [18, 19]. Essential oils (EOs) are volatile, aromatic mixtures, consisting principally of terpenes and phenylpropane derivatives [20]. Oregano oils are essential oils obtained from oregano plant. The major components of oregano oils are carvacrol and thymol that constitute about 78 to 82% of the total oils [21]. It has been suggested that the oregano essential oil (OEO) has antimicrobial, antioxidant [22], and improving growth performance properties [23].

Based on their positive effects, benzoic acid,* bacillus* coagulans, and oregano oils are considered to be good potential alternatives for in-feed antibiotic growth promoter. However, the experimental results of these additives vary widely and the effect of a single additive is limited. In recent years, organic acids, probiotics, and essential oils combined supplementation in animal diets have received attention due to potential “synergistic” and “additive” benefits on growth performance under normal physiological conditions [24, 25]. However, there are few reports about the effects of benzoic acid,* bacillus* coagulans, and oregano oils combined supplementation on growth performance and intestinal health of weaned piglets under ETEC challenge.

Therefore, in this study, we used the ETEC-challenged weaned piglet model [26] to evaluate whether benzoic acid,* bacillus* coagulans, and oregano oil combined supplementation could protect growth performance by attenuating diarrhoea and intestinal injury and to examine the underlying mechanism.

## 2. Materials and Methods

### 2.1. Materials

Benzoic acid (VevoVitall) was purchased from the DSM (China) Company Limited (purity, 99.9%);* Bacillus* coagulans was provided by Sanzheng Group (Kunming, China) at a density of 5 × 10^9^ CFU/g; Oregano oil (free-flowing powder) was provided by Kemin Industries (Zhuhai, China); the major active components are carvacrol and thymol (contained a minimum of 22 g/kg carvacrol and a minimum of 11 g/kg thymol); defatted rice bran and silica were used as carriers.

### 2.2. Animal, Diets, and Experimental Design

The experimental protocol for the present study was approved by the Animal Care and Use Committee of Sichuan Agricultural University. In a 26-day study, a total of 30 crossbred (Duroc × Landrace × Yorkshire) piglets (initial body weight of 7.64 ± 0.46 kg) were randomly assigned to six treatments (n 5) based on their initial body weight. Pigs were individually housed in the metabolism cage (1.5 m × 0.7 m × 1.0 m) of two environmentally controlled nursery rooms (25-28°C) and* ad libitum *access to feed and water. The basal diet (Table 1) was formulated to meet or exceed National Research Council recommended nutrient requirements (NRC, 2012) for 7-25 kg piglets [27]. The experimental diets consisted of corresponding additive products replacing equivalent maize in basal diet.

The six treatment groups were as follows: (1) nonchallenged control group (CON: pigs fed basal diet and challenged with the sterile physiological saline); (2) ETEC-challenged control group (ETEC: pigs fed basal diet and challenged with enterotoxigenic* Escherichia coli*); (3) AT group (pigs fed basal diet with 20 g/t colistin sulfate + 40 g/t bacitracin zinc and challenged with enterotoxigenic* Escherichia coli*); (4) AB group (pigs fed basal diet with 3000 g/t benzoic acid + 400 g/t* bacillus *coagulans and challenged with enterotoxigenic* Escherichia coli*); (5) AO group (pigs fed basal diet with 3000 g/t benzoic acid + 400 g/t oregano oil and challenged with enterotoxigenic* Escherichia coli*); (6) ABO group (pigs fed basal diet with 3000 g/t benzoic acid + 400 g/t* bacillus *coagulans + 400 g/t oregano oil and challenged with enterotoxigenic* Escherichia coli*). On d 22 of the experimental period, all pigs except in CON were orally administered 3 × 10^11^ CFU ETEC (O149, K88 and K91, kindly provided by the College of Veterinary Medicine, Sichuan Agricultural University, China. Appropriate concentration: 1 × 10^9^ CFU/mL) and the CON group pigs were orally administered an equal volume of sterile physiological saline. The dose of ETEC was chosen according to our preliminary studies, which showed that piglets were induced diarrhoea significantly. The pigs in CON and pigs challenged with ETEC were housed separately to prevent cross-contamination. Two rooms were cleaned and disinfected, with similar conditions.

After ETEC infusion, the feces of all pigs were observed. Fecal consistency was scored as follows: 0, normal; 1, pasty; 2, semiliquid; and 3, liquid. Pigs with daily fecal consistency scores of ≥2 were considered to have diarrhoea [28]. Diarrhoea incidence (%) = the number of diarrhoea piglets in each pen × diarrhoea days / (the number of piglets of each pen × test days) × 100. Diarrhoea percentage (%) = the number of diarrhoea piglets of each treatment during the trial period / (the number of piglets of each treatment) × 100. Body weight and feed intake were measured at 08.00 hours on days 1, 22, and 27 to calculate average daily weight gain (ADG), average daily feed intake (ADFI), and the ratio of feed / gain (F/G).

### 2.3. Sample Collection and Preparation

On d 27, following weighing, bloods were sampled from the anterior vena cava and centrifuged at 3500 g for 10 min. The serum samples were stored at −80°C until analysis. After bleeding, all pigs were anesthetized by electric shock and then euthanized by exsanguinations. Then, the small intestine was removed, and the jejunum was quickly isolated. Approximately 20 cm of jejunal tissue sample was removed from the middle portion of jejunum and flushed with ice-cold saline to recover mucosa. The jejuna mucosa was sequentially collected by scraping the jejunal wall with a glass microscope slide. Samples were frozen in liquid nitrogen and stored at −80°C for real-time quantitative PCR and ELISA analysis. Approximately 3 g of the digesta from the jejunum and cecum were kept in sterile tubes separately and immediately frozen at −80°C for microbial DNA analysis.

### 2.4. Serum LPS Concentration and Diamine Oxidase Activity

The concentration of serum LPS was measured using the commercially available enzyme-linked immunosorbent assay (ELISA) kits from Xinle Co. Ltd. (Shanghai, China) according to the manufacturer's instructions. Serum diamine oxidase (DAO) activity was examined using the commercially available enzyme-linked immunosorbent assay (ELISA) kits from Nuoyuan Co. Ltd. (Shanghai, China) according to the manufacturer's instructions.

### 2.5. Serum and Jejunal Mucosal Cytokines Concentration, and Jejunal Mucosal sIgA Concentration

Serum TNF-*α*, IL-1*β*, IL-6, and IL-10 concentrations and the concentrations of TNF-*α*, IL-1*β*, IL-6, IL-10, and sIgA in jejunal mucosa of weaned pigs were determined using the commercially available enzyme-linked immunosorbent assay (ELISA) kits from Xinle Co. Ltd. (Shanghai, China) according to the manufacturer's instructions.

### 2.6. mRNA Expression Analysis by Real-Time PCR

Total RNA was extracted from the jejunal mucosa using TRIzol reagent (TaKaRa Biotechnology Co, Ltd, Dalian, China) following the manufacturer's instructions. The concentration and purity of RNA were analyzed spectrophotometrically (Beckman Coulter DU800; Beckman Coulter Inc.), considering the ideal absorbance ratio (1.8 ≤ A260/280 ≤ 2.0). The integrity of RNA was checked by electrophoresis on a 1.5% agarose gel. The RNA samples were reverse transcribed into complementary DNA using the PrimeScripte RT reagent kit (Takara) according to the manufacturer's instructions. The complementary DNA was diluted and used as a PCR template to evaluate gene expression. The primers were synthesized commercially by TaKaRa Biotechnology (Dalian) Co, Ltd. (Dalian, China), which were listed in Table 2. Quantitative real-time PCR was performed by conventional PCR on the option DNA Engine (Bio-Rad). The RT-PCR used SYBR Premix Ex TaqTM kits (TaKaRa) under the following conditions: predenaturation at 95°C for 30s and forty cycles of denaturation at 95°C for 5 s, annealing temperature (Table 2) for 30 s, and extension at 72°C for 60 s. A dissociation curve was constructed at the end of the reaction to ensure that only one amplification was formed. The expression of the target genes relative to housekeeping gene (glyceraldehyde-3-phosphate dehydrogenase; GAPDH) was analyzed with the previous method [29]. Each standard and sample was run simultaneously in triplicate on the same PCR plate, and their average value expressed as the number of copies was used for statistical analysis.

### 2.7. Bacterial DNA Isolation and Microbial Real-Time Quantitative PCR

Bacterial DNA in jejunal and cecal digesta were extracted by using the Stool DNA Kit (Omega Bio-tek) according to the manufacturer's instruction. The microbial real-time quantitative PCR was determined as described previously [30]. Briefly, the number of total bacteria was analyzed by real-time quantitative PCR using SYBR Premix Ex Taq reagents (TaKaRa Biotechnology (Dalian) Co, Ltd., Dalian, China) and CFX-96 Real-Time PCR Detection System (BioRad Laboratories, Richmond, CA), and the numbers of* Bacillus, Lactobacillus, E. coli*, and* Bifidobacterium* were analyzed by real-time quantitative PCR using PrimerScript TM PCR kit (Perfect Real-Time; TaKaRa Biotechnology (Dalian) Co, Ltd, Dalian, China) and CFX-96 Real-Time PCR Detection System (Bio-Rad Laboratories, Richmond, CA) as previously described [30]. All primers and probes (Table 3) were purchased from TaKaRa Biotechnology (Dalian) Co, Ltd. (Dalian, China). For the quantification of bacteria in the test samples, specific standard curves were generated by constructing standard plasmids as presented by Chen et al [30]. In addition, bacterial copies were transformed (log_10_) before statistical analysis.

### 2.8. Statistical Analysis

All data were presented as means with their pooled standard errors and analyzed by one-way analysis of variance. Student's t-test was used to detect differences in means between the control group and the ETEC group. Duncan's multiple-comparison test was used to detect differences among the means of the ETEC-challenged treatment groups. Differences were considered as significant at* P *< 0.05 and 0.05 ≤* P *≤ 0.1 were discussed as tendency. All statistical analyses were performed with commercially available statistics software (SPSS 17.0).

## 3. Results

### 3.1. Growth Performance and Diarrhoea

The results of growth performance and diarrhoea were shown in Table 4. During 1 to 21 d (prechallenge) of the trial, AB and ABO supplementation significantly increased ADG and decreased F/G compared with ETEC group (*P*<0.05). During 22 to 26 d (postchallenge), ETEC challenge resulted in a 23.28% reduction of ADG and a 21.54% increasing of F/G compared with CON group (*P*>0.05). However, AO and ABO supplementation significantly prevented the reducing of ADG (*P*<0.05) and the increasing of F/G (*P*<0.05) induced by ETEC. The diarrhoea incidence and diarrhoea percentage of piglets in ETEC group were higher than that in CON group (*P*<0.05). Compared with ETEC group, dietary AT, AB, AO, and ABO supplementation decreased the diarrhoea incidence (*P*<0.05); furthermore, AB and ABO decreased the diarrhoea percentage of piglets (*P*<0.05).

### 3.2. Inflammatory Cytokines Concentrations in Serum

Concentrations of inflammatory cytokines in serum were presented in Table 5. Compared with CON group, ETEC challenge increased serum TNF-*α* and IL-1*β* concentrations (*P*<0.05). However, dietary AB, AO, and ABO inhibited the enhancing of serum TNF-*α* concentration induced by ETEC (*P*<0.05). Meanwhile, lower serum IL-1*β* concentration was observed in AO and ABO groups compared with ETEC group (*P*<0.05). No difference was observed on serum IL-6 and IL-10 concentrations among treatments (*P*>0.05).

### 3.3. Inflammatory Cytokines and sIgA Concentrations in Jejunal Mucosa

As shown in Table 6, ETEC challenge increased TNF-*α* and IL-1*β* concentrations and decreased sIgA concentration in jejunal mucosa (*P*<0.05). ABO supplementation inhibited the elevation of TNF-*α* and IL-1*β* concentrations and the reduction of sIgA concentration in jejunal mucosa induced by ETEC (*P*<0.05). Meanwhile, the IL-1*β* concentration of the jejunal mucosa in AO group was lower than that in ETEC group (*P*<0.05). However, no difference was observed on IL-6 and IL-10 concentrations among treatments (*P*>0.05).

### 3.4. Bacteria Populations in Jejunal and Cecal Digesta

As shown in Table 7, ETEC challenge increased* Escherichia coli* population (*P*<0.05) and trended to decrease* Lactobacillus* and* Bacillus* populations (*P*<0.10) in cecal digesta. However, compared with ETEC group, AT increased* Lactobacillus *and* Bacillus* populations in cecal digesta (*P*<0.05), AB decreased* Escherichia coli* population in jejunal digesta and increased* Bacillus* population in cecal digesta (*P*<0.05), and AO increased* Lactobacillus* population and decreased* Escherichia coli* population in jejunal digesta and increased* Lactobacillus, Bacillus,* and total bacteria populations in cecal digesta (*P*<0.05). Furthermore, ABO increased* Lactobacillus* and* Bifidobacterium *populations in jejunal digesta and increased* Lactobacillus, Bacillus*, and total bacteria populations in cecal digesta and decreased* Escherichia coli* populations in jejunal and cecal digesta (*P*<0.05).

### 3.5. Serum LPS Concentration and DAO Activity and Jejunal Mucosal Barrier Junction Related Gene Expression

As shown in Table 8, ETEC challenge increased LPS concentration in serum (*P*<0.05). Lower LPS concentration was observed in AT, AB, AO, and ABO groups compared with ETEC group (*P*<0.05). The serum LPS concentration in AB, AO, and ABO groups was also lower than that in AT group (*P*<0.05). Compared with CON group, ETEC challenge downregulated mRNA abundances of Claudin-1*(P*<0.10) and Mucin2 (*P*<0.05) in jejunal mucosa. However, AT diet inhibited the downregulation of Occludin and ZO-1 mRNA expressions (*P*<0.05), AO suppressed the reduction of Claudin-1 mRNA expression, and ABO prevented the downregulation of Claudin-1 and Occludin mRNA abundances induced by ETEC (*P*<0.05). Furthermore, the mRNA abundance of Mucin2 was upregulated in AT, AB, AO, and ABO groups compared with ETEC group (*P*<0.05).

### 3.6. Jejunal Mucosal mRNA Expression of TLR4 and NODs and Their Downstream Signals

As shown in Table 9, ETEC challenge increased the mRNA abundances of TLR4, MYD88, P_38_MAPK, and NF-*κ*Bp65 in jejunal mucosa of piglets compared with CON group (*P*<0.05). The increasing of P_38_MAPK mRNA abundance in jejunal mucosa induced by ETEC was downregulated by AB treatment (*P*<0.05). AO supplementation suppressed the elevation of NF-*κ*Bp65 mRNA expression induced by ETEC (*P*<0.05). Additionally, the mRNA expressions of TLR4, MYD88, IRAK1, P_38_MAPK, NF-*κ*Bp65, and TNF-*α* in jejunal mucosa were decreased in ABO group compared with ETEC group (*P*<0.05). ETEC challenge increased jejunal mucosal RIPK2 mRNA expression (*P*<0.05) and trended to increase NOD2 mRNA expression (*P*<0.10) relative to CON group, however, which was attenuated by ABO treatment (*P*<0.05). Similarly, both AB and AO suppressed the ETEC-induced upregulation of RIPK2 mRNA expression in jejunal mucosa (*P*<0.05).

## 4. Discussion

In this study, we used ETEC challenge model to investigate the potential protective effects of dietary benzoic acid,* bacillus* coagulans, and oregano oil combined supplementation on growth performance and intestinal barrier of piglets. We found that ETEC challenge resulted in a 23.28% reduction of ADG and a 21.54% increasing of F/G of piglets compared with CON group, which was consistent with previous observations that the growth performance of weaned piglets is impaired by an ETEC challenge [31, 32]. The compromised growth performance was probably due to the diversion of available nutrients away from growth to support immune-related processes and synthesis of various mediators such as cytokines [33, 34]. On the other hand, the ETEC-induced intestinal dysfunction will further impair the digestion and absorption of nutrients. In addition, our results found that ETEC challenge significantly increased the diarrhoea incidence of piglets and induced 100% diarrhoea of pigs fed with basal diet, which supported the ETEC challenge model [35, 36]. Interestingly, the compromised growth performance and the increased diarrhoea of piglets challenged with ETEC were alleviated by dietary AO and ABO, which indicated that dietary benzoic acid,* bacillus* coagulans, and oregano oil combined supplementation could improve growth performance and alleviate diarrhoea of piglets. Previous studies have also revealed that a combination of benzoic acid with essential oils could improve growth performance and intestinal health of turkey poultry [37]. Similar results also reported that dietary benzoic acid, essential oils, and* enterococcus faecium* SF68 combined supplementation could improve growth performance of piglets [38].

Microflora in the gastrointestinal tract plays a crucial role in the physiological and immunological organ development of the host animals [39]. Benzoic acid,* bacillus* coagulans, and oregano oil could improve the intestinal ecosystem of animals via promoting the growth of beneficial bacteria species and suppressing the growth of potential pathogenic bacterial species [13, 40, 41]. In the present study, ETEC challenge decreased* Lactobacillus *and* Bacillus* populations and increased* Escherichia coli* population in cecal digesta, which was in agreement with previous observations that ETEC challenge decreased intestinal beneficial bacterial and increased harmful bacterial numbers [42]. At the same time, dietary supplementation with different combinations of benzoic acid,* bacillus* coagulans, and oregano oil, or antibiotics can prevent the ETEC-induced imbalance of flora in cecal and jejunal digesta, which was evidenced by the improving in* Lactobacillus* or* Bacillus* populations and the decreasing in* Escherichia* coli population of jejunal or cecal digesta. These results were consistent with the lower diarrhoea incidence of piglets in AT, AB, AO, and ABO groups. In addition, Konstantinov et al. indicated that a healthy and stable microflora prevented the development of intestinal diseases and resulted in good performance [43]. This phenomenon may be associated with the changes in the intestinal mucosa barrier integrity [44].

The intestinal mucosa is not only the major site for nutrients digestion and absorption but also plays a key role in host defense against pathogen infection. If the permeability of intestinal barrier increased, which would lead to the impaired epithelial cell function and the invasion of pathogenic bacteria, it would finally cause intestinal inflammation. Intestinal barrier function can be commonly assessed by some indices such as LPS concentration, DAO activity, and tight junction proteins. LPS is the major component of outer membranes of gram-negative bacteria, mainly expressed in small intestine and rarely in serum under normal circumstances. When intestinal barrier integrity was damaged, tissue LPS levels decreased and serum LPS levels increased [45]. Our results showed that ETEC challenge increased serum LPS concentration of piglets, while dietary AT, AB, AO, and ABO inhibited this increase, indicating their ability to protect the barrier integrity of the intestinal mucosa. Tight junction (TJ) proteins [occludin, claudin, and intracellular plaque proteins (ZO and cingluin)], which participate in tight junction structural integrity via binding to the actin cytoskeleton, are considered as major constituents of tight junctions and important regulators of paracellular permeability [46]. In the current experiment, compared with ETEC group, dietary AT increased the mRNA expressions of Occludin and ZO-1, AO increased Claudin-1 mRNA expression, and ABO increased the mRNA expressions of Claudin-1 and Occludin in jejunal mucosa of weaned piglets. Besides TJ proteins, mucus layer is the first barrier of defense encountered by intestinal bacteria and mucins are the primary constituent of the mucus layer [47]. In addition, Mucin1 and Mucin2 are the major mucin proteins in small intestine. In the present study, ETEC challenge decreased Mucin2 mRNA abundance. However, dietary AT, AB, AO, and ABO alleviated the decrease in Mucin2 mRNA abundance induced by ETEC. Our results suggested that benzoic acid,* bacillus* coagulans, and oregano oil combined supplementation could help restore the intestinal barrier integrity and function of piglets following ETEC challenge; however, the underlying mechanisms are still not clear.

As the crucial component of the humoral immunity, sIgA plays an important role in protecting the intestinal epithelium from enteric toxins and pathogenic microorganisms [48]. In the present study, dietary ABO prevented the reduction of jejunal mucosal sIgA concentration induced by ETEC. The result suggested that dietary benzoic acid,* bacillus* coagulans, and oregano oil supplementation together could improve the humoral immunity of intestinal mucosa in piglets challenged with ETEC.

Cytokines are an important part of the body's cellular immune, which play a critical role in lymphocyte development and subsequent functional activity of the peripheral immune compartment [49]. TNF-*α*, IL-1*β*, and IL-6 are the important proinflammatory cytokines, which regulate the host immunity against multiple pathogens through immune cell differentiation, proliferation, and apoptosis [50]. However, excessive and long-term production of proinflammatory cytokines might lead to body and gut damage [51]. In the present study, ETEC challenge increased serum TNF-*α* and IL-1*β* concentrations, which was consistent with the previous reports [52, 53]. However, AB supplementation prevented the increase in serum TNF-*α* concentrations and AO and ABO alleviated the elevation of serum TNF-*α* and IL-1*β* levels. The results suggested that benzoic acid,* bacillus* coagulans, and oregano oil combined supplementation could improve the immune function of piglets to resist the attack of ETEC. Besides the important roles in immunity, cytokines also demonstrated to affect tight junction [54]. Studies have shown that the function and permeability of the intestine may be regulated by a network of multiple cytokines, including TNF-*α*, IL-1*β*, and IL-10, through modulation of tight junctions proteins and regulation of junction assembly [55]. Proinflammatory cytokines could induce disruption of tight junction, which led to increased intestinal permeability, whereas anti-inflammatory cytokines tended to protect the intestinal integrity [56]. In the present study, consistent with mucosal injury caused by ETEC challenge, increased TNF-*α* and IL-1*β* concentrations in jejunal mucosa were observed. The results were consistent with previous reports that overproduction of proinflammatory cytokines had a negative influence on gut integrity and epithelial function [57]. However, dietary ABO supplementation decreased TNF-*α* and IL-1*β* concentrations and AO decreased IL-1*β* concentration in jejunal mucosa compared with ETEC group. These results indicated that dietary AO and ABO may improve intestinal integrity partially by suppressing ETET-induced proinflammatory cytokine production.

To elucidate the molecular mechanism by which benzoic acid,* bacillus* coagulans, and oregano oil combined supplementation attenuate intestinal inflammatory response, we investigated the response of two inflammatory signaling pathways, including transmembrane TLRs and intracellular NODs [58, 59]. Current research has demonstrated that activation of TLRs and NODs signaling is associated with multilayered inflammatory intestinal diseases [60, 61]. TLRs are an ancient conserved family of pattern-recognition receptors which play a critical role in recognizing microbial pathogens and modulating antimicrobial host defense [62]. Among this family, TLR4 is the best-characterized member. TLR4 is responsible for recognizing endotoxin or LPS from gram-negative bacteria and initiating the systemic inflammatory response syndrome [63]. LPS is mainly recognized through the TLR4/MD2/CD14 complex [64]. When engaged by LPS, TLR4 transmits a signal that is passed onwards by a cascade of MyD88, IRAK1, and TRAF6 and finally triggers the activation of multiple intracellular signaling pathways, predominantly including NF-*κ*B pathway, as well as MAPKs pathways, which include the Jun N-terminal kinase, ERK1/2, and p38 [64]. Activation of these intracellular signaling pathways further leads to expression and release of proinflammatory cytokines such as IL-1*β*, IL-6, and TNF-*α* [65]. In the current experiment, intestinal mRNA upregulation of TLR4 and its downstream signaling molecules, including MYD88, P_38_MAPK, and NF-*κ*Bp65, was observed, which was consistent with the intestinal inflammation caused by ETEC challenge. Interestingly, the increasing of P_38_MAPK mRNA abundance in jejunal mucosa caused by ETEC was reduced by dietary AB and the upregulation of NF-*κ*Bp65 mRNA expression in jejunal mucosa was inhibited by dietary AO. Additionally, dietary ABO suppressed the mRNA expressions of TLR4, MYD88, IRAK1, P_38_MAPK, NF-KBp65,and TNF-*α* in jejunal mucosa of weaned piglets challenged with ETEC. So far, the research on probiotics and essential oils modulating TLR signaling in the neuroendocrine system is limited. Eunok et al. demonstrated that M1201* Bacillus* polyfermenticus ameliorated colonic inflammation and suppressed mucosal apoptosis in experimental colitis models via TLR2 and TLR4 signaling pathways [66]. Lee et al. reported that leaf essential oil significantly lowered peripheral levels of IL-1*β* and TNF-*α* and inhibited the mRNA expressions of TLR4 and MYD88 in endotoxin-injected mice [67]. Based on these data, the protective effects of dietary ABO on intestinal barrier integrity might be associated with decreasing proinflammatory cytokines production via inhibition of TLR4/NF-*κ*Bp65 and P_38_MAPK signaling pathway.

Apart from TLRs, another family of pattern-recognition receptors, cytoplasmic NODs, also play key roles in recognition of PAMPs and regulation of host innate immune response [68]. Among the NOD family, NOD1 and NOD2 are the best-characterized members. Similar to TLR4, NOD1 and NOD2 also can activate NF-*κ*B via an adaptor molecule, RIPK2, resulting in transcriptional upregulation of proinflammatory cytokine genes [64]. Though LPS is not a ligand for NOD1 and NOD2, NOD1 and NOD2 have been shown to be activated by LPS through TLR4 and TNF-*α* [69]. In the present experiment, similar to TLR4 signaling pathway, we also found that ETEC challenge increased NOD2 and RIPK2 mRNA expressions in jejunal mucosa of weaned piglets. In contrast, the NOD2 and RIPK2 mRNA expressions in jejunal mucosa were lower in ABO group than that of ETEC group. Additionally, the decreased mRNA abundance of RIPK2 in jejunal mucosa was observed in AB and AO group. In this study, it is possible that the protective effects of dietary ABO on intestinal barrier integrity are also associated with the reduction of proinflammatory cytokines production via inhibition of NOD2/NF-*κ*Bp65 signaling pathway.

## 5. Conclusions

In conclusion, benzoic acid,* bacillus *coagulans, and oregano oil combined supplementation could improve growth performance and alleviate diarrhoea of piglets challenged with ETEC via improving intestinal mucosal barrier integrity, which was possibly associated with the improvement of intestinal microbiota and the reduction of proinflammatory cytokines production via inhibition of TLR4 and NOD2 signaling pathways. The combination of 3000 g/t benzoic acid + 400 g/t* bacillus* coagulans + 400 g/t oregano oil showed better effects than other treatments in improving growth performance and intestinal health of piglets, which could be used as a viable substitute for antibiotic.

## Figures and Tables

**Table 1:** Ingredients composition and nutrients levels of the basal diet (as-fed basis).

Ingredient composition	Content (%)	Nutrient levels^3^	Content
Maize (7.8%CP)	29.80	Digestible energy, MJ/kg	14.72
Extruded maize	29.85	Crude protein %	19.13
Fishmeal (62% CP)	4.50	Ca %	0.75
Whey powder (3% CP)	6.00	Total P %	0.56
Sucrose	3.00	Available P %	0.37
Soybean meal (46% CP)	10.00	Digestible Lys %	1.30
Soybean protein concentrate	6.40	Digestible Met %	0.41
Extruded soybean	6.70	Digestible Met + Cys %	0.70
Soybean oil	1.70	Digestible Thr %	0.79
L-Lysine-HCl	0.26	Digestible Trp %	0.22
L-Threonine	0.02		
DL-Methionine	0.09		
L-Tryptophan	0.01		
Choline chloride	0.15		
NaCl	0.20		
CaCO_3_	0.60		
CaHPO_4_	0.37		
Vitamin premix^1^	0.05		
Mineral premix^2^	0.30		
Total	100.00		

^1^Vitamin premix provided the following per kg of diets: Vitamin A, 9000 IU; Vitamin D3, 3000 IU; Vitamin E, 20 IU; Vitamin K3, 3.0 mg; Vitamin B1, 1.5 mg; Vitamin B2, 4.0 mg; Vitamin B6, 3.0 mg; Vitamin B12, 0.02 mg; Niacin, 30 mg; Pantothenic, 15 mg; Folic acid, 0.75 mg; Biotin, 0.1 mg.

^2^Mineral premix provided the following per kg of diets: Fe, 100 mg; Cu, 150 mg; Mn, 20 mg; Zn, 100 mg; I, 0.3 mg; Se, 0.3 mg.

^3^Nutrients levels were calculated values.

**Table 2:** Primer sequences used for real-time PCR.

Primer	Primer sequence (5′ –3′)	Anneal temperature (°C)	Product length (bp)	GeneBank accession No.
GAPDH	F:TGAAGGTCGGAGTGAACGGAT R:CACTTTGCCAGAGTTAAAAGCA	55.7	114	NM_001206359.1
Claudin-1	F: GCCACAGCAAGGTATGGTAAC R: AGTAGGGCACCTCCCAGAAG	59.0	140	FJ873109.1
Occludin	F: CTACTCGTCCAACGGGAAAG R:ACGCCTCCAAGTTACCACTG	59.0	158	NM_001163647.2
ZO-1	F: CAGCCCCCGTACATGGAGA R: GCGCAGACGGTGTTCATAGTT	59.0	114	XM_005659811
Mucin1	F: GTGCCGCTGCCCACAACCTG R:AGCCGGGTACCCCAGACCCA	59.0	141	XM_001926883.4
Mucin2	F:GGTCATGCTGGAGCTGGACAGT R:TGCCTCCTCGGGGTCGTCAC	59.0	181	XM_003122394.1
TLR4	F: TTACAGAAGCTGGTTGCCGT R:TCCAGGTTGGGCAGGTTAGA	63.3	152	GQ304754
CD14	F: CCTCAGACTCCGTAATGTG R: CCGGGATTGTCAGATAGG	59.0	180	AB267810
MYD88	F: CCATTCGAGATGACCCCCTG R: TAGCAATGGACCAGACGCAG	59.0	183	NM_001099923.1
TRIF	F: CAAGTGGAGGAAGGAACAGG R: CAACTGCGTCTGGTAGGACA	59.0	139	XM_003362039.1
IRAK1	F: CAAGGCAGGTCAGGTTTCGT R:TTCGTGGGGCGTGTAGTGT	55.7	115	XM_003135490.1
TRAF6	F: CAAGAGAATACCCAGTCGCACA R: ATCCGAGACAAAGGGGAAGAA	55.7	122	NM-001105286.1
P_38_MAPK	F:AGTTGAAGCTCATTTTAAGACTCGT R: AGTTCATCTTCGGCATCTGGG	55.7	117	XM_001929490.5
NF-*κ*Bp65	F: GTGTGTAAAGAAGCGGGACCT R: CACTGTCACCTGGAAGCAGAG	55.7	139	EU399817.1
IL-1*β*	F: CAGCTGCAAATCTCTCACCA R: TCTTCATCGGCTTCTCCACT	55.7	112	NM_214055.1
TNF-*α*	F: CGTGAAGCTGAAAGACAACCAG R: GATGGTGTGAGTGAGGAAAACG	55.7	121	NM_214022.1
NOD1	F: CTGTCGTCAACACCGATCCA R: CCAGTTGGTGACGCAGCTT	55.7	57	AB187219.1
NOD2	F: GAGCGCATCCTCTTAACTTTCG R: ACGCTCGTGATCCGTGAAC	55.7	66	AB195466.1
RIPK2	F: CAGTGTCCAGTAAATCGCAGTTG R: CAGGCTTCCGTCATCTGGTT	59.0	206	XM_003355027.1

GAPDH: glyceraldehyde 3-phosphate dehydrogenase; TLR4: toll-like receptor 4; MYD88: myeloid differentiation factor 88; IRAK1: IL-1 receptor-associated kinase 1; TRAF6: TNF receptor-associated factor 6; P_38_MAPK: P_38_ mitogen-activated protein kinase; NF-*κ*Bp65: nuclear factor-*κ*B p65; IL-1*β*: interleukin-1*β*; TNF-*α*: tumor necrosis factor-*α*; NOD: nucleotide-binding oligomerization domain protein; RIPK2: receptor-interacting serine/threonine-protein kinase 2.

**Table 3:** Primer and probe sequences used for real-time PCR.

Items	Primer and probe sequence (5′ –3′)	Anneal temperature (°C)	Product length (bp)
Total bacteria	F: ACTCCTACGGGAGGCAGCAG R: ATTACCGCGGCTGCTGG	57.9	200
*Lactobacillus*	F: GAGGCAGCAGTAGGGAATCTTC R: CAACAGTTACTCTGACACCCGTTCTTC P: AAGAAGGGTTTCGGCTCGTAAAACTCTGTT	53.0	126
*Bifidobacterium*	F: CGCGTCCGGTGTGAAAG R: CTTCCCGATATCTACACATTCCA P: ATTCCACCGTTACACCGGAA	57.9	121
*Bacillus*	F:GCAACGAGCGCAACCCTTGA R:TCATCCCCACCTTCCTCCGGT P: CGGTTTGTCACCGGCAGTCACCT	53.0	92
*Escherichia coli*	F: CATGCCGCGTGTATGAAGAA R: CGGGTAACGTCAATGAGCAAA P: AGGTATTAACTTTACTCCCTTCCTC	53.0	96

**Table 4:** Effects of dietary benzoic acid, *bacillus *coagulans, and oregano oil combined supplementation on growth performance and diarrhoea of weaned piglets challenged with *Escherichia coli.*

Items	CON	ETEC	AT	AB	AO	ABO	SEM	*P1∗*	*P2* ^†^
1-21 d									
ADFI (g)	407.50	405.59	444.32	486.36	477.14	484.82	12.42	0.963	0.178
ADG (g)	244.29	229.37^a^	272.38^ab^	337.14^b^	291.90^ab^	310.95^b^	11.99	0.664	0.038
F/G	1.72	1.78^b^	1.65^ab^	1.45^a^	1.66^ab^	1.58^a^	0.03	0.640	0.016
22-26 d									
ADFI (g)	558.76	490.60	524.48	561.48	609.24	615.24	17.81	0.259	0.252
ADG (g)	292.00	224.00^a^	274.00^ab^	306.00^ab^	352.00^b^	356.00^b^	14.43	0.181	0.046
F/G	1.95	2.37^b^	2.01^ab^	1.86^ab^	1.74^a^	1.76^a^	0.08	0.237	0.131
Diarrhoea incidence (%)	0.00	44.00^#b^	24.00^a^	8.00^a^	8.00^a^	4.00^a^	3.58	<0.001	0.002
Diarrhoea percentage (%)	0.00	100.00^#b^	60.00^ab^	20.00^a^	40.00^ab^	20.00^a^	10.20	<0.001	0.054

ADFI: average daily feed intake; ADG: average daily weight gain; F/G: feed/gain.

In the same row, different superscript letters show significant difference among ETEC-challenged groups (*p *< 0.05).

*∗P1 *was used to determine the response to ETEC challenge, CON *v.* ETEC.

^†^ *P2* was used to determine the response to benzoic acid, *bacillus* coagulans, and oregano oil combined supplementation among ETEC-challenged piglets.

**Table 5:** Effects of dietary benzoic acid, *bacillus* coagulans, and oregano oil combined supplementation on serum TNF-*α*, IL-1*β*, IL-6, and IL-10 concentrations of piglets challenged with *Escherichia coli*.

Items	CON	ETEC	AT	AB	AO	ABO	SEM	*P1∗*	*P2* ^†^
TNF-*α* (ng/L)	203.49	223.45^#b^	203.55^ab^	194.23^a^	195.30^a^	183.46^a^	3.67	0.001	0.036
IL-1*β* (ng/L)	11.30	19.82^#c^	17.99^c^	17.09^bc^	14.19^ab^	12.39^a^	0.70	0.002	0.001
IL-6 (ng/L)	67.56	77.93	74.40	73.16	72.50	70.12	1.32	0.133	0.395
IL-10 (pg/ml)	190.65	172.85	175.69	175.70	187.02	192.44	4.61	0.348	0.623

TNF-*α*: tumor necrosis factor-*α*; IL-1*β*: interleukin-1*β*; IL-6: interleukin-6; IL-10: interleukin-10.

^#^Significantly different from CON group (*p*< 0.05). In the same row, different superscript letters show significant difference among ETEC-challenged groups (*p *< 0.05).

*∗P1* was used to determine the response to ETEC challenge, CON *v. *ETEC.

^†^ *P2* was used to determine the response to benzoic acid,* bacillus *coagulans, and oregano oil combined supplementation among ETEC-challenged piglets.

**Table 6:** Effects of dietary benzoic acid, *bacillus *coagulans, and oregano oil combined supplementation on inflammatory cytokines and sIgA concentrations in jejunal mucosa of weaned piglets challenged with *Escherichia coli*.

Items	CON	ETEC	AT	AB	AO	ABO	SEM	*P1∗*	*P2* ^†^
TNF-*α* (ng/L)	96.67	126.72^#b^	101.95^ab^	106.99^ab^	104.30^ab^	91.85^a^	3.87	0.050	0.158
IL-1*β* (ng/L)	14.11	19.77^#b^	15.80^ab^	15.03^ab^	13.89^a^	13.24^a^	0.71	0.017	0.068
IL-6 (ng/L)	59.52	66.87	62.82	63.27	59.06	51.43	2.84	0.476	0.617
IL-10 (pg/ml)	163.08	134.96	175.23	168.09	176.50	‘167.63	7.17	0.186	0.488
sIgA (ug/ml)	46.30	36.92^#a^	45.98^ab^	50.05^ab^	48.61^ab^	57.48^b^	2.14	0.025	0.131

TNF-*α*: tumor necrosis factor-*α*; IL: interleukin; sIgA: secretion immunoglobulin A.

^#^Significantly different from CON group (*p*< 0.05). In the same row, different superscript letters show significant difference among ETEC-challenged groups (*p*< 0.05).

*∗P1* was used to determine the response to ETEC challenge, CON *v. *ETEC.

^†^ *P2* was used to determine the response to benzoic acid, *bacillus *coagulans, and oregano oil combined supplementation among ETEC-challenged piglets.

**Table 7:** Effects of dietary benzoic acid, *bacillus *coagulans, and oregano oil combined supplementation on intestinal bacteria in the jejunal and cecal digesta of piglets challenged with *Escherichia coli *(log_10_(copies/g)).

Items	CON	ETEC	AT	AB	AO	ABO	SEM	*P1∗*	*P2* ^†^
jejunum									
*Lactobacillus*	7.25	6.89^a^	7.38^ab^	7.33^ab^	7.53^b^	7.62^b^	0.08	0.127	0.055
*Bacillus*	10.99	10.88	10.95	10.99	11.01	11.00	0.02	0.148	0.466
*Bifidobacterium*	6.64	6.59^a^	7.28^ab^	7.23^ab^	7.13^ab^	7.37^b^	0.10	0.887	0.185
*Escherichia coli*	7.48	8.46^b^	7.82^ab^	6.98^a^	7.25^a^	6.98^a^	0.18	0.151	0.054
Total bacteria	9.29	9.18	9.41	9.41	9.39	9.42	0.06	0.618	0.736
Cecum									
*Lactobacillus*	8.40	7.70^a^	8.20^b^	8.09^ab^	8.29^b^	8.32^b^	0.08	0.065	0.040
*Bacillus*	9.84	9.60^a^	9.85^b^	10.04^b^	9.82^b^	9.88^b^	0.04	0.083	0.008
*Bifidobacterium*	7.88	7.74	7.79	8.14	7.81	8.03	0.07	0.333	0.494
*Escherichia coli*	8.51	9.56^#b^	8.87^ab^	8.76^ab^	8.75^ab^	8.35^a^	0.15	0.001	0.285
Total bacteria	11.29	11.18^a^	11.39^ab^	1.38^ab^	11.41^b^	11.49^b^	0.03	0.407	0.055

^#^Significantly different from CON group (*p*< 0.05). In the same row, different superscript letters show significant difference among ETEC-challenged groups (*p*< 0.05).

*∗P1* was used to determine the response to ETEC challenge, CON *v. *ETEC.

^†^ *P2* was used to determine the response to benzoic acid,* bacillus *coagulans, and oregano oil combined supplementation among ETEC-challenged piglets.

**Table 8:** Effects of dietary benzoic acid, *bacillus* coagulans and oregano oil combined supplementation on serum LPS concentration and DAO activity and jejunal mucosal barrier junction related gene expression of weaned piglets challenged with *Escherichia coli*.

Items	CON	ETEC	AT	AB	AO	ABO	SEM	*P1∗*	*P2* ^†^
Serum									
LPS EU/L	111.51	186.74^#c^	158.08^b^	113.16^a^	104.67^a^	99.69^a^	6.40	<0.001	<0.001
DAO U/mL	31.85	35.02	37.43	37.18	36.01	33.29	0.69	0.269	0.412
Jejunal mucosa									
Claudin-1	1.00	0.58^a^	1.12^ab^	1.19^ab^	1.36^b^	1.51^b^	0.09	0.059	0.045
Occludin	1.00	0.69^a^	1.47^b^	1.18^ab^	1.09^ab^	1.45^b^	0.08	0.156	0.019
ZO-1	1.00	0.78^a^	1.29^b^	1.09^ab^	1.11^ab^	1.13^ab^	0.05	0.249	0.062
Mucin1	1.00	0.82	1.40	1.45	1.39	1.51	0.10	0.190	0.353
Mucin2	1.00	0.60^#a^	1.05^b^	1.17^b^	1.07^b^	1.18^b^	0.06	0.037	0.008

^#^Significantly different from CON group (*p*< 0.05). In the same row, different superscript letters show significant difference among ETEC-challenged groups (*p*< 0.05).

*∗P1* was used to determine the response to ETEC challenge, CON *v. *ETEC.

^†^ *P2* was used to determine the response to benzoic acid, *bacillus *coagulans, and oregano oil combined supplementation among ETEC-challenged piglets.

**Table 9:** Effects of dietary benzoic acid,* bacillus *coagulans and oregano oil combined supplementation on mRNA expression of TLR4 and NODs and their downstream signaling molecules in jejunal mucosa of weaned piglets challenged with *Escherichia coli*.

Items	CON	ETEC	AT	AB	AO	ABO	SEM	*P1∗*	*P2* ^†^
TLR4	1.00	2.52^#b^	2.00^ab^	1.64^ab^	1.86^ab^	1.49^a^	0.15	0.047	0.331
CD14	1.00	1.63	1.51	1.48	1.49	1.57	0.15	0.116	0.999
MYD88	1.00	1.55^#b^	1.45^ab^	1.27^ab^	1.31^ab^	1.06^a^	0.06	0.008	0.120
TRIF	1.00	0.92	1.04	0.96	1.06	0.89	0.06	0.769	0.863
IRAK1	1.00	1.30^b^	1.10^ab^	0.96^ab^	0.93^ab^	0.85^a^	0.05	0.157	0.098
TRAF6	1.00	1.23	1.20	1.00	1.02	1.00	0.04	0.053	0.280
P_38_MAPK	1.00	1.52^#b^	1.46^b^	1.04^a^	1.17^ab^	1.02^a^	0.06	0.050	0.049
NF-*κ*Bp65	1.00	1.46^#b^	1.20^ab^	1.14^ab^	1.03^a^	1.06^a^	0.05	0.020	0.099
IL-1*β*	1.00	1.48	0.94	0.90	1.11	0.93	0.10	0.354	0.466
TNF-*α*	1.00	1.43^b^	0.92^ab^	1.08^ab^	1.29^ab^	0.80^a^	0.08	0.221	0.121
NOD1	1.00	1.02	0.92	0.82	0.86	0.63	0.07	0.961	0.470
NOD2	1.00	1.51^b^	1.23^ab^	1.19^ab^	1.27^ab^	1.06^a^	0.07	0.077	0.117
RIPK2	1.00	1.61^#b^	1.37^ab^	1.17^a^	1.26^a^	1.14^a^	0.05	0.010	0.028

TLR4: toll-like receptor 4; MYD88: myeloid differentiation factor 88; IRAK1: IL-1 receptor-associated kinase 1; TRAF6: TNF receptor-associated factor 6; P_38_MAPK: P_38_ mitogen-activated protein kinase; NF-*κ*Bp65: nuclear factor-*κ*B p65; IL-1*β*: interleukin-1*β*; TNF-*α*: tumor necrosis factor-*α*; NOD: nucleotide-binding oligomerization domain protein; RIPK2: receptor-interacting serine/threonine-protein kinase 2.

^#^Significantly different from CON group (*p *< 0.05).In the same row, different superscript letters show significant difference among ETEC-challenged groups (*p *< 0.05).

*∗P1* was used to determine the response to ETEC challenge, CON *v. *ETEC.

^†^ *P2* was used to determine the response to benzoic acid, *bacillus* coagulans, and oregano oil combined supplementation among ETEC-challenged piglets.

## Data Availability

The data used to support the findings of this study are included within the article.
